# *Wolbachia* Inhibits Binding of Dengue and Zika Viruses to Mosquito Cells

**DOI:** 10.3389/fmicb.2020.01750

**Published:** 2020-08-04

**Authors:** Peng Lu, Qiang Sun, Ping Fu, Kuibiao Li, Xiao Liang, Zhiyong Xi

**Affiliations:** ^1^ Department of Microbiology and Molecular Genetics, Michigan State University, East Lansing, MI, United States; ^2^ School of Basic Medical Sciences, Guizhou Medical University, Guiyang, China; ^3^ Guangzhou Center for Disease Control and Prevention, Guangzhou, China

**Keywords:** *Wolbachia*, dengue, Zika, viral entry, mosquito

## Abstract

As traditional approaches to the control of dengue and Zika are insufficient, significant efforts have been made to develop utilization of the endosymbiotic bacterium *Wolbachia* to reduce the ability of mosquitoes to transmit pathogens. Although *Wolbachia* is known to inhibit flaviviruses in mosquitoes, including dengue virus (DENV) and Zika virus (ZIKV), it remains unclear how the endosymbiont interferes with viral replication cycle. In this study, we have carried out viral binding assays to investigate the impact of the *Wolbachia* strain *w*AlbB on the attachment of DENV serotype 2 (DENV-2) and ZIKV to *Aedes aegypti* Aag-2 cells. RNA interference (RNAi) was used to silence a variety of putative mosquito receptors of DENV that were differentially regulated by *w*AlbB in Aag-2 cells, in order to identify host factors involved in the inhibition of viral binding. Our results showed that, in addition to suppression of viral replication, *Wolbachia* strongly inhibited binding of both DENV-2 and ZIKV to Aag-2 cells. Moreover, the expression of two putative mosquito DENV receptors – dystroglycan and tubulin – was downregulated by *w*AlbB, and their knock-down resulted in the inhibition of DENV-2 binding to Aag-2 cells. These results will aid in understanding the *Wolbachia*-DENV interactions in mosquito and the development of novel control strategies for mosquito-borne diseases.

## Introduction

Dengue virus (DENV), a member of the family *Flaviviridae*, is the causative agent of dengue fever, dengue hemorrhagic fever, and dengue shock syndrome. As a major public health problem, with approximately 2.5 billion people at risk of pathogen transmission, DENV causes up to 50 million infections annually, in over 100 endemic countries, with 22,000 deaths mainly among children (Bhatt et al., 2013). Zika virus (ZIKV) is another flavivirus, which, in 2016, the World Health Organization (WHO) declared a Public Health Emergency of International Concern, because of its outbreak in the Americas and the widespread microcephaly and other neurological disorders it caused. Both DENV and ZIKV are transmitted to humans by *Aedes* mosquitoes, including the two species *Aedes aegypti* and *Aedes albopictus*. Lack of effective vaccines and antiviral therapies means that vector control is the primary intervention tool, which has been insufficient to prevent the global spread of dengue (Guzman et al., 2010). In order to meet the challenge of controlling DENV and ZIKV, innovative approaches – including *Wolbachia*-based replacement and suppression of mosquito vector populations – are currently under development for disease control (Hoffmann et al., 2011; Zheng et al., 2019).

Flaviviruses, including DENV and ZIKV, are enveloped positive-strand RNA viruses with a genome of approximately 11 kilobases, which have a single open reading frame encoding three structural proteins – capsid (C), membrane (M), and envelope (E) protein – and seven non-structural proteins (NS1, NS2A, NS2B, NS3, NS4A, NS4B, and NS5; Mukhopadhyay et al., 2005; Harris et al., 2006). The viral life cycle begins with the binding of virions to their cellular receptors on the surface of susceptible host cells (Yazi Mendoza et al., 2002; Thepparit and Smith, 2004; Reyes-Del Valle et al., 2005; Mercado-Curiel et al., 2006), followed by entrance into the host cells through receptor-mediated endocytosis (Acosta et al., 2008; Miller et al., 2008; van der Schaar et al., 2008), then fusion of the viral membrane with the endosome membrane, and subsequent delivery of the nucleocapsid into the cell cytoplasm (Heinz and Allison, 2003; Bressanelli et al., 2004). Following uncoating of the nucleocapsid in the cell cytoplasm, replication and translation of the viral RNA begin. Assembly of immature virions occurs on the surface of the host cell endoplasmic reticulum (ER), with newly formed nucleocapsids budding into the ER lumen (Welsch et al., 2009; Apte-Sengupta et al., 2014). Subsequently, the immature virions are transported through the trans-Golgi network (TGN), where they mature and form infectious particles (Zybert et al., 2008). Finally, the mature flaviviruses are released from the host cell by exocytosis.

*Wolbachia* are maternally transmitted intracellular symbiotic bacteria that are estimated to infect >65% of insect species and a large number of other arthropods, including ticks and mites, as well as filarial nematodes (Hilgenboecker et al., 2008). Through cytoplasmic incompatibility (Werren, 1997), *Wolbachia* can rapidly invade and become fixed in mosquito populations not already infected with the same *Wolbachia* stain (Xi et al., 2005; Hoffmann et al., 2011). Furthermore, different *Wolbachia* strains have been observed to induce resistance to DENV in mosquitoes (Moreira et al., 2009; Bian et al., 2010; Walker et al., 2011; Ford et al., 2019), with the strength of viral inhibition depending on the density of *Wolbachia* (Osborne et al., 2009, 2012; Lu et al., 2012; Chrostek et al., 2013). This *Wolbachia*-mediated pathogen interference also has a broad spectrum, being effective against a variety of RNA viruses, including ZIKV, West Nile virus (WNV), and yellow fever and chikungunya viruses, as well as eukaryotic parasites, such as *Plasmodium* and filarial nematodes (Kambris et al., 2009; Moreira et al., 2009; Glaser and Meola, 2010; van den Hurk et al., 2012; Bian et al., 2013; Hussain et al., 2013). Although the detailed mechanism(s) underlying viral interference are not well understood, it is believed that both immune priming and metabolic alterations of the host contribute to pathogen resistance (Pan et al., 2012; Caragata et al., 2013). For example, *Wolbachia* induces the production of reactive oxygen species in both naturally-infected and artificially-transinfected insect hosts, which can trigger either direct and/or indirect antiviral responses (Brennan et al., 2008; Pan et al., 2012; Wong et al., 2015). *Wolbachia* also perturbs host metabolic pathways/networks (Caragata et al., 2013; Melnikow et al., 2013), which may interfere with host factors required for completion of the viral life cycle (Guo et al., 2010). In addition, genetic variation in certain host factors has been observed to affect the strength of *Wolbachia*-mediated viral blocking in mosquitoes (Ford et al., 2019). However, the impact of *Wolbachia* on the flavivirus life cycle has not yet been fully characterized. Previous studies have found that the *Wolbachia* strain *w*Stri inhibited both ZIKV entry into *A. albopictus* cells and replication of the viral genome (Schultz et al., 2018). By contrast, the *Wolbachia* strain *w*Mel was not observed to inhibit DENV binding or entry to *A. aegypti* Aag-2 cells (Thomas et al., 2018). The *Wolbachia* strain *w*MelPop was reported to enhance replication of the WNV genome, but it reduced production of secreted virus in the Aag-2 cell line (Hussain et al., 2013). However, a reduction of WNV and DENV replication, rather than enhancement, was observed in both *w*Mel-infected and *w*AlbB-infected Aag-2 cells, respectively (Lu et al., 2012; Thomas et al., 2018). Thus, further studies are needed to clarify how the virus life cycle is affected by the presence of *Wolbachia* in host cells, and the universality of these impacts are among both different flaviviruses and *Wolbachia* strains. As different host factors participate in each stage of the virus life cycle (Kuadkitkan et al., 2010; Colpitts et al., 2011; Munoz Mde et al., 2013), knowledge of how the viral life cycle is affected by *Wolbachia* could provide important insights allowing further dissection of *Wolbachia* – flavivirus interactions in the mosquito host, thus facilitating the development of *Wolbachia*-mosquito symbioses with the greatest possible viral blocking.

We have previously shown that *w*AlbB induces strong resistance to the DENV serotype 2 (DENV-2) in the Aag-2 cell line (Lu et al., 2012) and that the strength of this viral inhibition depends on the density of *w*AlbB within host cells (Lu et al., 2012). DENV-2 is eliminated at a high density of *Wolbachia* in host cells, while both virus and the endosymbiont coexist in the cytoplasm of mosquito cells if *w*AlbB is present at a low density (Lu et al., 2012). In this study, in order to better understand how DENV is inhibited by *w*AlbB, we have focused on the impact of *w*AlbB on DENV-2 life cycle. Our results show that *w*AlbB prevents the intracellular accumulation of viral genome copies in Aag-2 cells by inhibiting the binding of both DENV-2 and ZIKV to Aag-2 cells and so prevents viruses from entering the next stage of their life cycle. Furthermore, we identify several mosquito host proteins bound by DENV, whose expression is downregulated by *w*AlbB and for which gene silencing is shown to interfere with viral binding in mosquito cells.

## Materials and Methods

### Cell and Viral Culture

The *A. aegypti* W-Aag-2 cell line was generated by transinfecting Aag-2 cells (Peleg, 1968) with *Wolbachia* using the shell vial technique, as previously described (Lu et al., 2012). The R-Aag-2 cell line was generated from W-Aag-2 by treatment of the latter with the antibiotic rifampicin (Lu et al., 2012). The W-Aag-2 and R-Aag-2 cell lines were maintained at 25°C in Schneider’s Drosophila Medium (Invitrogen) supplemented with 10% (v/v) heat-inactivated fetal bovine serum (FBS) and 1% penicillin/streptomycin (Life Technologies) and were passaged at 1:5 dilution every 6–7 days.

The New Guinea C (NGC) strain of DENV-2 was grown in W-Aag-2 and R-Aag-2 cells, as previously described (Sim and Dimopoulos, 2010). Briefly, cells were seeded in a 48-well plate to a confluency of 80%. W-Aag-2 and R-Aag-2 monolayers were then infected with DENV-2 at the desired multiplicity of infection (MOI) of DENV-2. Plates were incubated at 25°C for the duration of the experiment. The ZIKV PRVABC59 strain, obtained from ATCC, was grown in Vero cells and cultured with Dulbecco’s Modified Eagle Medium (10% FBS) at 37°C with 5% CO2, and the titer was measured by plaque assay.

### DENV-2 and ZIKV Binding Assays

Binding assays were carried out to characterize the attachment of DENV and ZIKV to W-Aag-2 and R-Aag-2 cells. Prior to the initiation of viral binding, the culture medium was removed and cells were washed with cold Schneider’s Drosophila Medium. Subsequently, viruses were overlain on the cell cultures and incubated with the cells for 1 h at 4°C, with either DENV-2 at an MOI of 1 or 10 or ZIKV at an MOI of 0.1. The cells were washed three times with cold phosphate-buffered saline (PBS) to remove any unbound virus, followed by the addition of 350 μl buffer RLT (QIAGEN) to each well for RNA extraction. The number of gene copies of bound DENV-2 and ZIKV were quantified by real-time PCR. For the DENV-2 assays, the incubation medium was collected for measurement of the titer of unbound viruses.

### RNA Extraction, cDNA Synthesis, and Quantitative Reverse Transcription Polymerase Chain Reaction

Total RNA was extracted from the cell lines using the RNeasy Mini Kit (QIAGEN), and then the cDNA transcript was produced using the QuantiTect Reverse Transcription Kit (QIAGEN). Real-time PCR was conducted using the QuantiTect SYBR Green PCR Kit (QIAGEN) and an ABI Prism 7900HT Sequence Detection System (Applied Biosystems). DENV-2 genomic RNA was measured by quantitative reverse transcription PCR (qRT-PCR) using primers directed to the *NS5* gene (Molina-Cruz et al., 2005). The copy numbers of both DENV-2 and ZIKV genomes were normalized using the host (*A. aegypti*) ribosomal protein S6 (*rps6*) gene. A standard curve was generated for each of the *NS5* and *rps6* genes by analyzing 10^1^–10^8^ copies/reaction of two different plasmids, containing a fragment of each gene (Lu et al., 2012). The number of genome copies of bound ZIKV was quantified by qRT-PCR using the primers ZIKV 835 and ZIKV 911c (Lanciotti et al., 2008). *Wolbachia*-regulated expression of mosquito DENV-binding proteins was assayed using the primers listed in Supplementary Table S1.

Tagged RT-PCR was used to specifically amplify the negative sense viral RNA by preventing false priming (Peyrefitte et al., 2003). The primer tagF 5'-CGGTCATGGTGGCGAATAAACAAGTAGAACAACCTGGTCCAT-3' was designed to contain the DENV-targeting sequence in its 3'-end and a 19-mer-long non-DENV sequence in its 5'-end. After RNA extraction, the RNAs were denatured at 65°C for 3 min in the presence of 20 pmol of tagF primer for the negative strand-specific reverse transcription. cDNA was synthesized without addition of the RT primer mix. Real-time PCR was performed with a forward primer Tag 5'-CGGTCATGGTGGCGAATAA-3' and a DENV-targeting reverse primer, as previously described (Molina-Cruz et al., 2005). The host *rps6* gene was used to normalize the cDNA template.

### Indirect Immunofluorescence Assay

Cells were seeded in an 8-well plate to a confluency of 80%. After the medium was removed, cells were washed with PBS, fixed with 4% formaldehyde solution for 15 min at room temperature, and then treated with 0.5% Triton X-100 in PBS for 5 min. Samples were incubated with 10% non-fat dry milk blocking solution at room temperature with gentle shaking for 1 h, followed by incubation with a rabbit anti-WSP primary antibody (GenScript) at 1:500 and Alexa Fluor 488-conjugated secondary antibody (Invitrogen) at 1:1000. After incubating with 0.1 μg/ml DAPI for 1 min, the samples were examined using an Olympus FluoView 1000 Laser Scanning Confocal Microscope.

### *Wolbachia* Quantitative PCR

Quantitative PCR (qPCR) was performed to measure the density of *Wolbachia* in W-Aag-2 cells, as described previously (Tortosa et al., 2008). In brief, genomic DNA was extracted and *w*AlbB was amplified with the forward primer 183F (5'-AAGGAACCGAAGTTCATG-3') and the reverse primer QBrev2 (5'-AGTTGTGAGTAAAGTCCC-3'), which are specific for the *Wolbachia* surface protein (*wsp*) gene. The *Wolbachia* genome copy was normalized with the host *rps6* gene.

### DENV-2 RNA Transfection

W-Aag-2 and R-Aag-2 cells were seeded in a 48-well plate for 24 h prior to transfection and were at a confluency of 70–80% at the time of transfection (1 × 10^5^ cells/well). DENV-2 RNA was extracted from virus-infected cell culture supernatant using RNeasy Mini Kit (QIAGEN). The infectious DENV RNA was transfected using TransIT-mRNA Transfection Kit (Mirus) according to the manufacturer’s instructions. Briefly, DENV-2 RNA (0.5 μg) was incubated with 1 μl messenger RNA (mRNA) Boost Reagent and 1 μl *TranIT*-mRNA Reagent in 26 μl Schneider’s Drosophila Medium for 5 min. The mixture was then transferred to the 48-well plate with W-Aag-2 or R-Aag-2 cells already grown to a 70–80% confluence (1 × 10^5^ cells/well) in 260 μl complete medium. Four hours later, the medium containing transfection reagent was removed and replaced with normal fresh culture medium. This latter time point was designated as 0 h post-transfection. Cell lysates were collected at 0 h, 4 h, 3 days, and 7 days post-transfection to measure the levels of total DENV-2 RNA (0 h) and negative strand RNA (4 h, 3 days, and 7 days).

### Plaque Assays for DENV-2 Virus Titration

DENV-2 titers were measured by plaque assays, as previously reported (Das et al., 2007; Bian et al., 2010). Briefly, C6/36 cells were seeded in the 48-well plate at a density of 4–8 × 10^4^ cells/well and maintained for 2–3 days at 32°C in 5% CO_2_. The virus-containing culture medium was serially diluted and inoculated into C6/36 cells. After incubation for 5 days, plaque forming units (PFUs) were measured in the plates by peroxidase immunostaining, using mouse hyperimmune ascitic fluid (specific for DENV-2; CDC) as the primary antibody and a goat anti-mouse horseradish peroxidase (HRP) conjugate as the secondary antibody.

### RNAi-Mediated Gene Silencing

Double-stranded RNA (dsRNA) was synthesized from PCR-amplified gene fragments using the MEGAscript T7 High Yield Transcription Kit (Ambion). The sequences of the primers are listed in Supplementary Table S2. Transfection of dsRNA was carried out using Attractene Transfection Reagent (Qiagen) according to the manufacturer’s instruction. Briefly, cells were seeded in the 48-well plate for 24 h prior to transfection. One microgram of dsRNA was incubated with 3.5 μl Attractene Transfection Reagent in 50 μl Schneider’s Drosophila Medium for 10–15 min at room temperature and then transferred to each well. Three days post-transfection, DENV-2 binding assays were then performed at 4°C on R-Aag-2 and W-Aag-2 cells with an MOI of 10. Gene silencing efficiency was determined by comparing the relative mRNA levels of the target gene after knockdown with its specific dsRNA and dsRNA of green fluorescent protein (dsGFP, the non-target control) using real-time PCR.

## Results

### *w*AlbB Inhibits Intracellular Accumulation of DENV-2 Genome Copies in Aag-2 Cells

**Figure 1:**
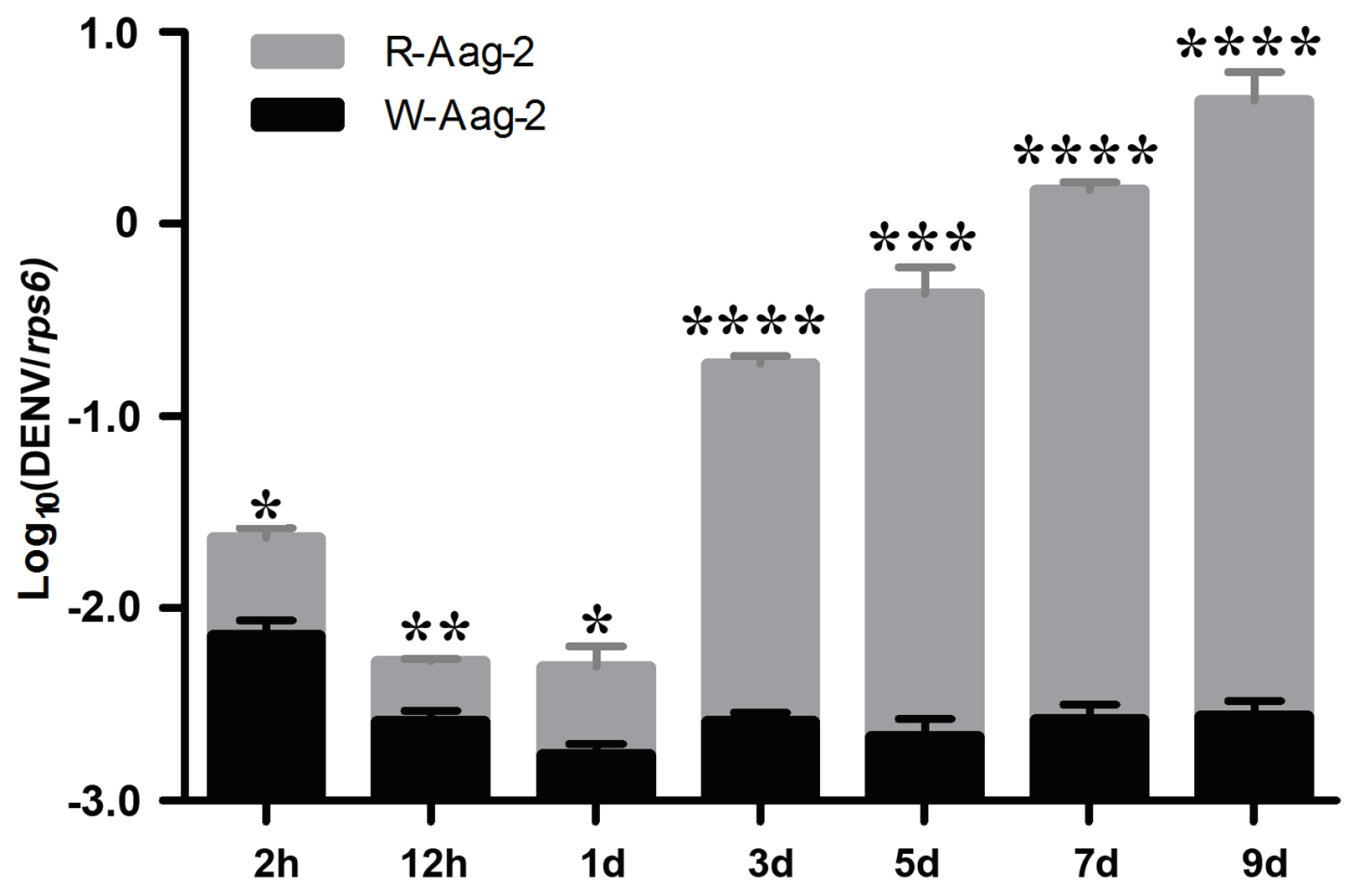
Comparison of dengue virus serotype 2 (DENV-2) dynamics in W-Aag-2 and R-Aag-2 cells. The copy number of DENV-2 genomic RNA was measured by quantitative reverse transcription PCR (qRT-PCR) in *Aedes aegypti* W-Aag-2 and R-Aag-2 cells at different times post-infection. Ribosomal protein S6 (*rps6*) gene was used as a host gene to normalize the data. Error bars are standard errors of the mean of three biological replicates for each cell line. ^*^p < 0.05; ^**^p < 0.01; ^***^p < 0.001; ^****^p < 0.0001; Student’s *t*-test.

We previously reported that the *Wolbachia* strain *w*AlbB induced density-dependent inhibition of DENV-2 in mosquito cells (Lu et al., 2012). In order to further investigate the dynamics of DENV suppression by *w*AlbB, we compared the number of genome copies of DENV-2 at various times post-infection in *w*AlbB-infected Aag-2 cells (W-Aag-2) and aposymbiotic cells (R-Aag-2, a cell line derived from W-Aag-2 cells through rifampicin treatment and used as a control). After both cells were infected with DENV-2 at an MOI of 1, the number of genome copies of DENV-2 was measured by qRT-PCR at seven different time points over the course of the 9-day experiment. Overall, the number of genome copies of DENV-2 was significantly lower in W-Aag-2 cells than R-Aag-2 cells at all seven of the time points assayed (Figure 1). At 2 h post-infection, the mean genome copy number of DENV-2 in R-Aag-2 cells was 3.2-fold higher than in W-Aag-2 cells, suggesting that *w*AlbB may interfere with early events in virus life cycle. The magnitude of variation in viral genome copies between two cell lines increased markedly from 3 dpi (i.e., when genome replication was first detectable in R-Aag-2 cells). Consequently, the viral copy number increased 72-, 200-, 574-, and 1,577-fold in R-Aag-2 cells relative to W-Aag2 cells at 3, 5, 7, and 9 dpi, respectively, indicating that *w*AlbB constantly and persistently inhibited intracellular accumulation of DENV genome copies in W-Aag-2 cells (Figure 1).

### *w*AlbB Inhibits Binding of DENV-2 and ZIKV to Aag-2 Cells

**Figure 2:**
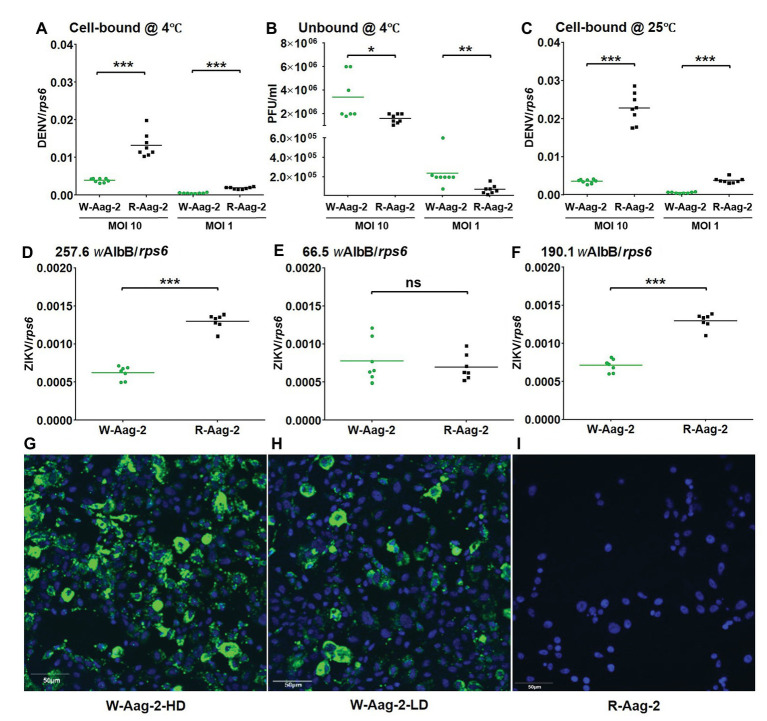
Inhibition of binding of DENV-2 and Zika virus (ZIKV) to mosquito cells by *w*AlbB. **(A)** Binding of DENV-2 to W-Aag-2 and R-Aag-2 cells at 4°C. W-Aag-2 and R-Aag-2 cells were incubated with DENV-2 at either 10 MOI or 1 MOI for 60 min at 4°C. Virus-cell binding was measured by qRT-PCR and normalized using *rps6*. **(B)** Infectivity to C6/36 cells lacking *Wolbachia* infection of unbound DENV-2 in the culture medium from W-Aag-2 and R-Aag-2 cells. After incubation at 4°C with W-Aag-2 and R-Aag-2 cells, viral titer in the culture medium was measured by plaque assay to determine the amount of unbound virus. **(C)** W-Aag-2 and R-Aag-2 cells were incubated with DENV-2 at an MOI of either 10 or 1 for 60 min at 25°C. **(D–F)** Binding of ZIKV to Aag-2 cells at a 0.1 MOI for 60 min at 4°C, with different *w*AlbB densities in W-Aag-2 cells as indicated. **(G–I)** Representative indirect immunofluorescence assay (IFA) pictures showing: **(G)** high *Wolbachia* density (W-Aag-2-HD) in W-Aag-2 cells, **(H)** low *Wolbachia* density (W-Aag-2-LD) in W-Aag-2 cells, and **(I)** the absence of *Wolbachia* in R-Aag-2 cells. Viral genomic copies were measured by qRT-PCR and normalized by *rps6*. Lines indicate the median value of the eight biological replicates ^*^p < 0.05; ^**^p < 0.01; ^***^p < 0.001; ns, not significant; Mann Whitney *U* test.

The observed viral inhibition at 2 h post-infection indicated that the viral interference might occur as early as virus binding to and/or entry into host cells. Thus, we tested whether *w*AlbB prevented DENV-2 from binding to Aag-2 cells. Both W-Aag-2 and R-Aag-2 cells were incubated with DENV-2 at an MOI of either 10 or 1 for 1 h at 4°C to allow virus binding to – but not penetration of – host cells (Salas-Benito and del Angel, 1997; Wei et al., 2003). Immediately, the challenged host cells were then washed three times with ice-cold medium, and the number of bound RNA copies of DENV-2 was determined by qRT-PCR. At an initial MOI of 10, the amount of DENV-2 bound to W-Aag-2 cells (0.0033 RNA copies per RNA copy of host *rps6*) was 3.9-fold lower than the amount bound to R-Aag-2 cells (0.013 copies per copy *rps6*; Figure 2A). This represents a 75% reduction in binding of DENV-2 to Aag-2 cells. Similar results were observed when mosquito cells were exposed to lower levels of virus. At an MOI of 1, the viral genome copy number (0.0006 copies per copy of *rps6*) was 3.2-fold lower in the W-Aag-2 cell line than R-Aag-2 cells (0.0019 copies per copy of *rps6*; Figure 2A). For further validation, we also measured, using plaque assays, the titers of unbound DENV in the incubation medium. Consistent with an inhibition of viral binding to cells, the unbound viral titer in the incubation medium of W-Aag-2 cells (3.4 × 10^6^ PFU/ml) was 2.1-fold higher than that of R-Aag-2 cells (1.6 × 10^6^ PFU/ml) at an MOI of 10 (Figure 2B). At an MOI of 1, a 3.3-fold increase in the unbound viral titer was also observed in the incubation medium of W-Aag-2 cells (2.4 × 10^5^ PFU/ml) compared to that of R-Aag-2 cells (7.2 × 10^4^ PFU/ml; Figure 2B). In order to test how viral infection was affected by a temperature that allowed DENV-2 to both bind and penetrate into host cells, we performed the same assays at 25°C. At an initial MOI of 10, the genome copy number of DENV-2 was 6.1-fold lower in W-Aag-2 cells (0.0036 copies per copy of *rps6*) than R-Aag-2 cells (0.022 copies per copy of *rps6*; Figure 2C). A similar reduction was also observed at an MOI of 1, where the copy numbers of viral genomic RNA were 6.6-fold lower in W-Aag-2 cells (0.00058 copies per copy of *rps6*) than R-Aag-2 (0.0038 copies per copy of *rps6*; Figure 2C). The moderate variation in viral inhibition between 4 and 25°C does not support the conclusion that the viral internalization process is affected by *Wolbachia* as the low number of internalized viruses may be a simple consequence of binding inhibition.

In order to know whether *w*AlbB-mediated inhibition of DENV-2 binding to mosquito cells might apply to other flaviviruses, we repeated the above viral binding assays using ZIKV. At an initial MOI of 0.1, the amount of ZIKV bound to W-Aag-2 cells (0.000621 copies per copy of *rps6*) was 2.1-fold lower than the amount bound to R-Aag-2 cells (0.001296 copies per copy of *rps6*; Figure 2D). Quantitative PCR showed that the estimated relative density of *w*AlbB in the W-Aag-2 cells during this experiment was 257.6 copies of the *wsp* gene per copy of host cell *rps6*. In order to test whether the observed inhibition of ZIKV was influenced by the density of *Wolbachia*, we performed the same experiment again using cells with a lower *Wolbachia* density (66.5 *wsp*/*rps6*), which were random cultures from the same W-Aag-2 cell line but had different *Wolbachia* densities in a particular generation of culture. No significant difference was observed in the amount of ZIKV bound to W-Aag-2 cells as compared to R-Aag2 cells (Figure 2E). Interestingly, the density of *w*AlbB increased to 190.1 *w*AlbB/RPS6 after six passages of the low density *Wolbachia* culture of the above Aag-2 cells, and significant inhibition of viral binding to W-Aag-2 cells was observed again following this increase in *w*AlbB density (Figure 2F). The above high and low densities of *w*AlbB in W-Aag-2 cells were also visualized using indirect immunofluorescence assay (IFA; Figures 2G–I). Overall, these results indicate that *w*AlbB needs a sufficiently high density to inhibit viral binding to mosquito cells.

### *w*AlbB Inhibits DENV Replication in Aag-2 Cells

**Figure 3:**
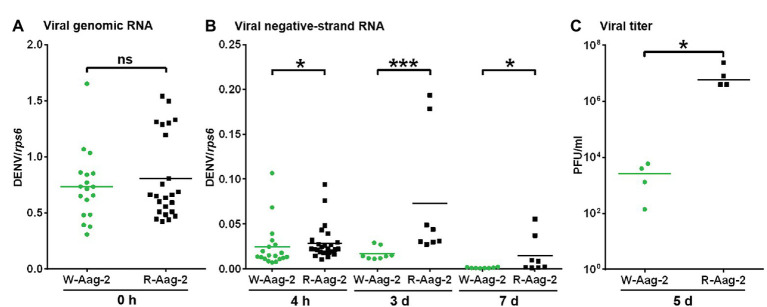
Suppression of DENV RNA replication by *w*AlbB in mosquito cells. **(A)** The number of copies of DENV-2 genomic RNA in W-Aag-2 and R-Aag-2 cells at 0 h post-transfection. **(B)** The number of copies of DENV-2 negative-strand RNA in W-Aag-2 and R-Aag-2 cells at various times post-transfection. Equivalent amounts of purified DENV-2 RNA were transfected into cells. Cells were sampled at 4 h and 3 and 7 days post-transfection. Negative-strand RNA was measured by Tag-PCR. **(C)** The titer of DENV-2 in the supernatant of W-Aag-2 and R-Aag-2 cells at 5 days post-transfection. Viral titer was measured by plaque assay. ^*^p < 0.05; ^***^p < 0.001; ns, not significant; Mann Whitney *U* test.

Given that the previous observation of *Wolbachia*-mediated viral interference is an accumulated outcome from viral binding and the other stages (Lu et al., 2012), we attempted to characterize the impact of *w*AlbB on virus replication alone. Thus, we delivered infectious DENV-2 RNA into host cells by transfection, in order to bypass the initial events in the life cycle of DENV infection – including binding, entry, nucleocapsid release, and uncoating – and then conducted tagged RT-PCR to measure negative-strand antigenomic RNA (Peyrefitte et al., 2003), a hallmark of active DENV replication (Tuiskunen et al., 2010). There was no significant difference in the amount of the viral genome (i.e., positive-strand RNA) in W-Aag-2 and R-Aag-2 cells at 0 h post-transfection (Figure 3A). However, we observed significantly lower copy numbers of viral negative-strand RNA at 4 h post-transfection in W-Aag-2 cells compared to R-Aag-2 cells (Figure 3B). This indicates that *w*AlbB inhibited virus genome replication by blocking synthesis of the viral negative-strand RNA. The copy number of viral negative-strand RNA was also measured at 3 and 7 days post-transfection. Again, there was a significantly lower copy number of viral negative-strand RNA in W-Aag-2 cells than R-Aag-2 cells at both time points (Figure 3B). In addition, the viral titer was also significantly lower in the supernatant of W-Aag-2 cells (2.8 × 10^3^ PFU/ml) than R-Aag2 cells (1.0 × 10^7^ PFU/ml) at 5 days post-transfection (Figure 3C). Overall, these observations indicate that *w*AlbB inhibits DENV-2 infection when the initial stages of the life cycle (i.e., binding, cell entry, and virion disassembly) are artificially by-passed using transfection.

### *w*AlbB Regulates Expression of Host Cell Proteins Bound by DENV

**Figure 4:**
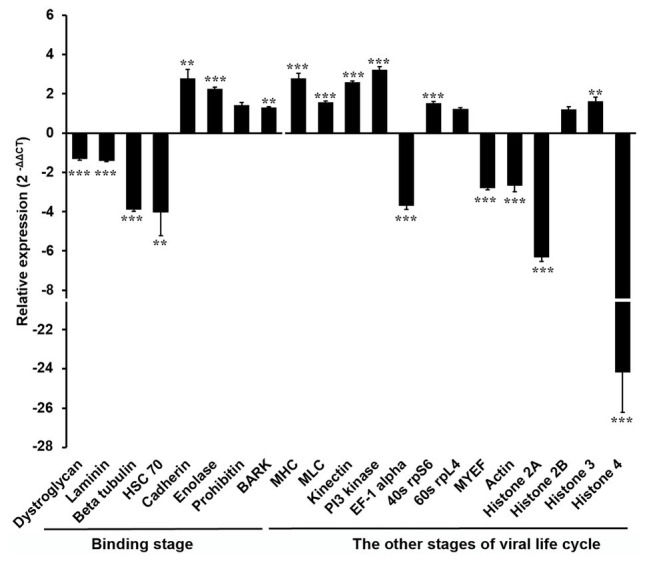
Differential expressions in W-Aag-2 cells compared to R-Aag-2 cells of 21 host genes encoding proteins bound by DENV. The 21 host proteins bound by DENV were grouped into those involved in either the binding stage or other stages of viral life cycle. qRT-PCR was used to quantify the expression of each gene relative to the *rps6* gene, which was used as an internal reference control to normalize the data. The 2^−ΔΔCT^ method was used to calculate the fold-change for each gene, and significance was determined based on comparison of ΔCT of each gene in W-Aag-2 and R-Aag-2 cells. Each gene has eight biological replicates. Mean ± SEM; ^**^p < 0.01; ^***^p < 0.001; Mann Whitney *U* test.

In order to explore the molecular mechanism by which the DENV life cycle is inhibited by *w*AlbB in mosquito cells, we selected 21 mosquito host proteins that were previously reported as being bound by DENV (Table 1; Kuadkitkan et al., 2010; Colpitts et al., 2011; Munoz Mde et al., 2013) and tested whether *w*AlbB influenced the expression of the genes encoding these proteins. The 21 mosquito DENV-binding proteins were classified into two broad groups based on whether they were cell surface membrane proteins likely to be involved in viral binding to the host cell or non-cell surface membrane proteins putatively involved in the other (i.e., intracellular) stages of the DENV life cycle. We measured and compared their transcription in W-Aag-2 and R-Aag-2 cells by qRT-PCR. As a result, we found that – with the exception of prohibitin (AAEL009345) – seven out of the eight host membrane proteins were regulated by *w*AlbB in W-Aag-2 cells (Figure 4). Among them, dystroglycan (AAEL013147), laminin (AAEL001477), beta-tubulin (AAEL002851), and HSC70 (DQ440299) were downregulated by *w*AlbB, while cadherin (AAEL001196), enolase (AAEL001668), and BARK (AAEL006868) were upregulated in W-Aag-2 cells. In addition, 11 out of the 13 mosquito non-cell surface membrane proteins possibly involved in other stages of DENV life cycle were also regulated by *w*AlbB (Figure 4). Remarkably, histone 4 (AAEL003863) was downregulated more than 24.2-fold in W-Aag-2 cells compared to R-Aag-2 cells (Figure 4).

**Table 1:** The 21 host proteins that were previously reported as being bound by DENV.

Gene ID	Gene name	Viral protein	Reference
AAEL003863	Histone 4	Capsid	(Colpitts et al., 2011)
AAEL015390	Histone 2A	Capsid	(Colpitts et al., 2011)
AAEL002851	Beta tubulin	E, NS2A	(Colpitts et al., 2011)
AAEL017096	EF-1 alpha	E, NS2A, NS4B	(Colpitts et al., 2011; Munoz Mde et al., 2013)
AAEL003670	Myelinprotein expression factor (MYEF)	NS2A	(Colpitts et al., 2011)
AAEL001928	Actin	Capsid, NS4B	(Colpitts et al., 2011)
AAEL001477	Laminin alpha-1, 2 chain	E	(Colpitts et al., 2011)
AAEL013147	Dystroglycan-like protein	E	(Colpitts et al., 2011)
AAEL015681	Histone 2B	Capsid	(Colpitts et al., 2011)
AAEL009994	60S ribosomal protein L4 (rpL4)	NS2A	(Colpitts et al., 2011)
AAEL006868	beta-adrenergic receptor kinase (BARK)	E	(Munoz Mde et al., 2013)
AAEL009345	Prohibitin	E	(Kuadkitkan et al., 2010)
AAEL000032	40S ribosomal protein S6 (*rpS6*)	NS2A	(Colpitts et al., 2011)
AAEL007439	Myosin light chain (MLC)	E	(Colpitts et al., 2011)
AAEL001668	Enolase	Capsid, E	(Colpitts et al., 2011; Munoz Mde et al., 2013)
AAEL001411	Myosin heavy chain (MHC)	E, NS2A	(Colpitts et al., 2011)
AAEL001196	Cadherin	E	(Colpitts et al., 2011; Munoz Mde et al., 2013)
AAEL000386	PI3 kinase	E	(Colpitts et al., 2011)
AAEL003594	Kinectin	Capsid	(Colpitts et al., 2011)
AAEL003827	Histone 3	Capsid	(Colpitts et al., 2011)
DQ440299	HSC70	E	(Paingankar et al., 2010)

### Silencing Membrane Binding Proteins Downregulated by *w*AlbB Results in Inhibition of DENV Binding to Aag-2 Cells

**Figure 5:**
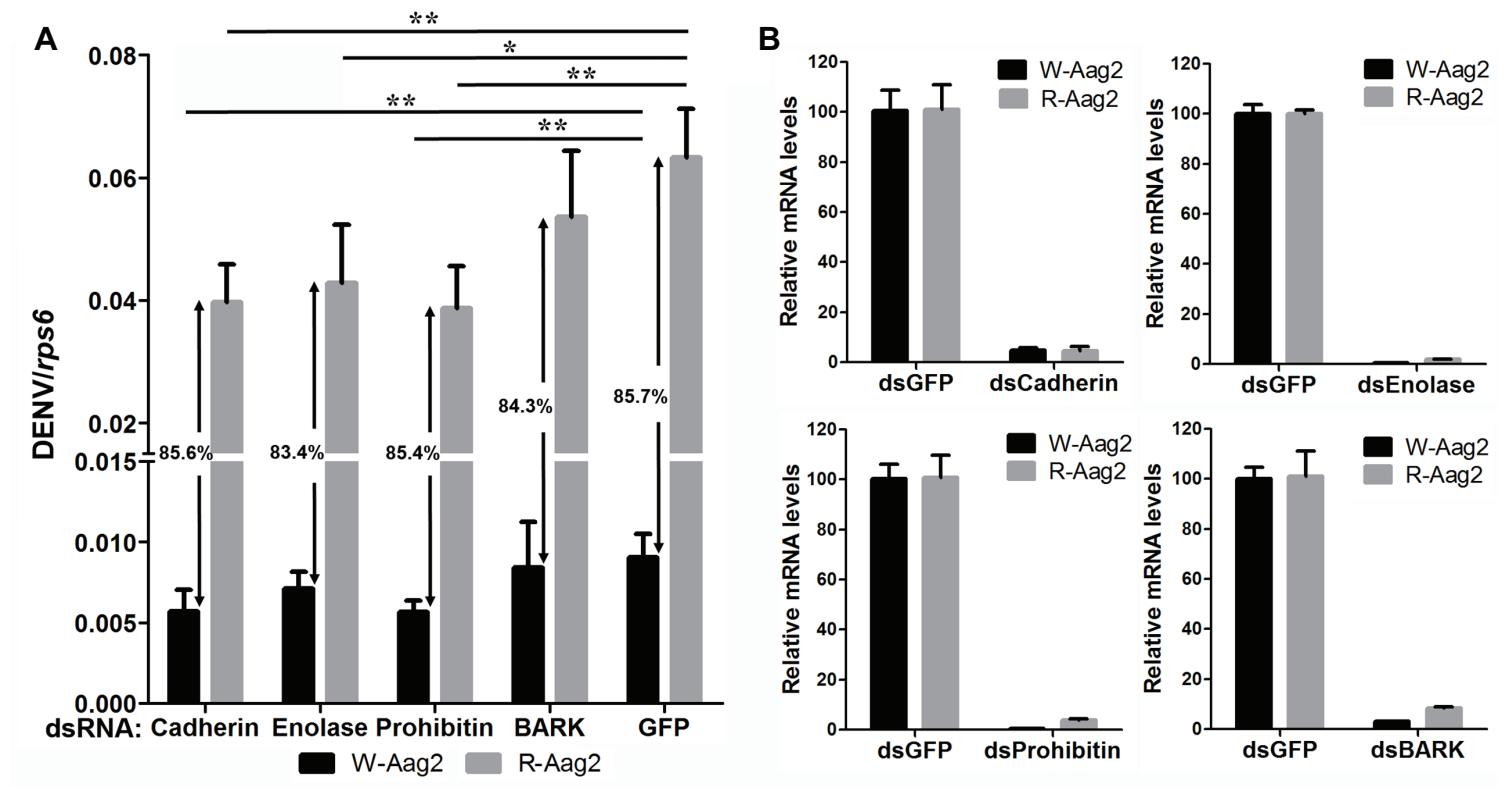
The contribution of *w*AlbB upregulating putative DENV mosquito receptors to viral binding interference. **(A)** Each gene was knocked down individually in both W-Aag-2 and R-Aag-2 cells, and its impact on viral binding was measured through comparison with their respective control groups (the dsGFP treatment). An 83–86% reduction in viral binding was consistently observed for W-Aag-2 cells compared to R-Aag-2 cells, regardless of which of these four genes was silenced. **(B)** Knockdown efficiency was measured by the relative messenger RNA (mRNA) levels of the target gene after its RNA interference (RNAi) silencing in both W-Aag-2 and R-Aag-2 cells as compared to the dsGFP treatment. Each treatment has six biological replicates. Mean ± SEM; ^*^p < 0.05, ^**^p < 0.01; ANOVA and Dunn’s multiple comparisons test.

**Figure 6:**
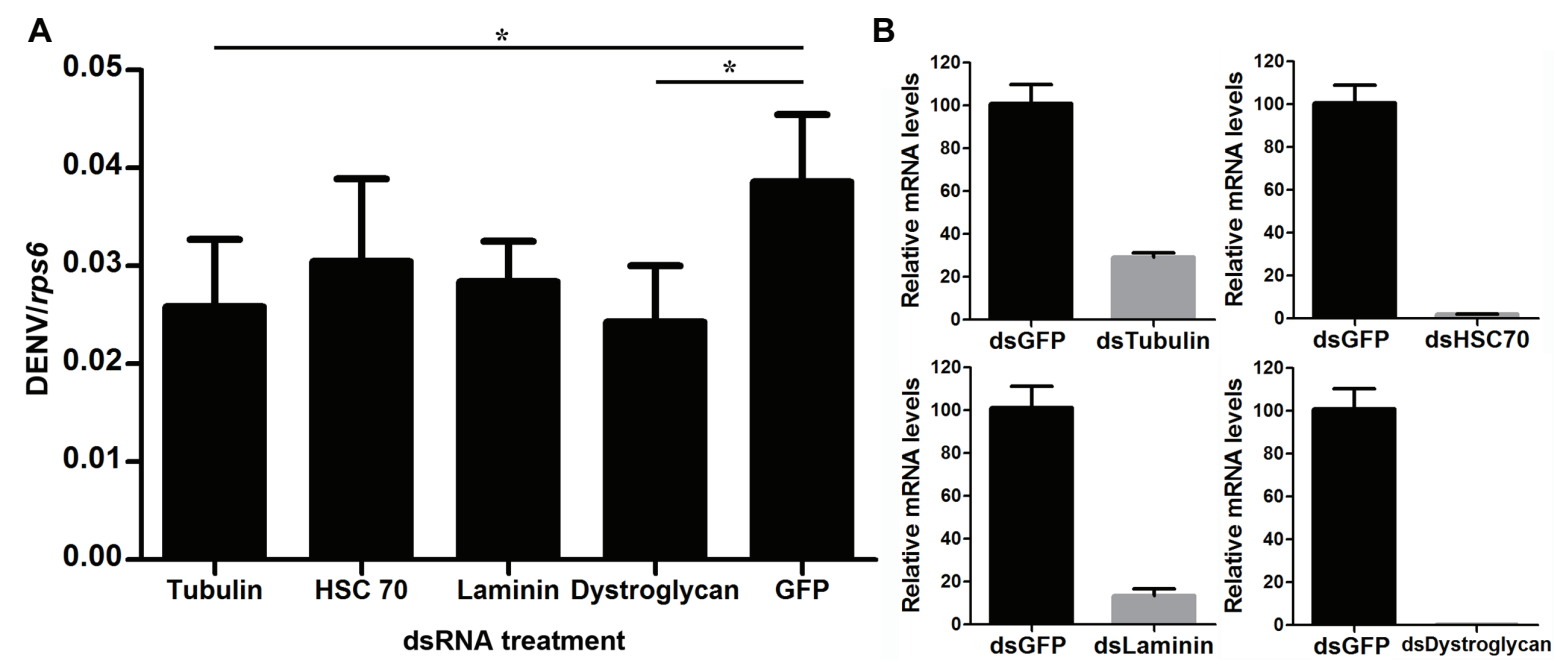
The role of *w*AlbB downregulating putative DENV mosquito receptors in viral binding interference. **(A)** Each gene was knocked down individually in R-Aag-2 cells, and its impact on viral binding was measured through comparison with the control group (with the dsGFP treatment). **(B)** Knock-down efficiency was measured by the relative mRNA levels of the target gene after its RNAi silencing as compared to the dsGFP treatment. Each treatment has six biological replicates. Mean ± SEM; ^*^p < 0.05; ANOVA and Dunn’s multiple comparisons test.

In order to examine how *w*AlbB inhibits DENV binding to Aag-2 cells, we used RNA interference (RNAi) to separately silence all eight of the mosquito host membrane proteins described above and then measured the copy number of DENV binding to host cells. For those genes that were upregulated by *w*AlbB, we knocked them down in both W-Aag-2 and R-Aag-2 cells using their respective dsRNAs and tested whether the inhibition of viral binding was compromised compared to the control groups in which RNAi was performed using dsGFP. Individual silencing of cadherin, enolase, BARK, and prohibitin had no effect on *w*AlbB-induced inhibition of viral binding to Aag-2 cells, and difference in viral binding between W-Aag-2 and R-Aag-2 cells stayed the same, regardless of which of these four genes was silenced (Figure 5A). These observations indicate that they were not involved in *w*AlbB-mediated viral binding interference even if they were upregulated by *w*AlbB. However, cadherin and prohibitin silencing resulted in significant reduction in the number of viruses binding to both W-Aag-2 and R-Aag-2 cells as compared to the control group (Figure 5A). A similar reduction was also observed in R-Aag-2 cells but not in W-Aag-2 cells, after enolase was knockdown. The above indicate that these genes regulate binding of DENV to Aag-2 cells, although unrelated to *w*AlbB-mediated binding inhibition effects. A similar and high knockdown efficiency was observed for all four of these genes in both W-Aag-2 and R-Aag-2 cells (Figure 5B). For the four membrane proteins that were downregulated by *w*AlbB, we individually knocked them down only in R-Aag-2 cells, in order to mimic the impact of *w*AlbB in the W-Aag-2 cells, and then tested whether the RNAi resulted in a similar inhibition of viral binding to host cells. Knockdown of dystroglycan and beta-tubulin led to a significant reduction in DENV-2 binding compared to the control using dsGFP treatment, while silencing of HSC70 and laminin had no effect (Figure 6A). Over 70% knockdown efficiency was achieved for all four genes encoding the *w*AlbB-downregulated membrane proteins (Figure 6B). These results suggest that downregulation of the transcription of dystroglycan and beta-tubulin by *w*AlbB may explain inhibition of DENV binding to Aag-2 cells.

## Discussion

*Wolbachia* has shown a great potential to be used as a biocontrol agent to prevent transmission of flaviviruses due to its ability both to suppress mosquito populations and to render them resistant to viruses. Understanding the mechanisms underlying this viral interference will help facilitate the development and improvement of *Wolbachia*-based strategies for disease control. In this work, we showed that the *Wolbachia* strain *w*AlbB was able to persistently inhibit the intracellular accumulation of DENV RNA in W-Aag-2 cells. *w*AlbB not only significantly inhibited the synthesis of viral negative-strand RNA but also decreased the number of DENV-2 virions binding to Aag-2 cells, thus preventing attachment of the virus to host cells. In addition, *w*AlbB also inhibited binding of ZIKV to Aag-2 cells, an effect that was dependent on *Wolbachia* density. Lastly, we provided evidence to show that *w*AlbB-induced downregulation of two potential mosquito dengue receptors – dystroglycan and tubulin – might contribute to inhibition of viral binding to mosquito cells.

The first step of DENV life cycle is binding to host cells before entry into them. Direct binding assays at a low temperature (4°C) have been previously used to prevent subsequent viral entry so that the binding step can be studied without consideration of the impact of the downstream steps (Salas-Benito and del Angel, 1997; Wei et al., 2003). Based on this approach, we found that the number of DENV attached to W-Aag-2 cells was significantly less than that to R-Aag-2 cells, at both high and low MOI. The magnitude of viral binding inhibition did not depend on the viral dose used to challenge the cells, and a 3–4 fold (or 68–75%) reduction in the number of virus binding to cells was observed at an MOI of both 10 and 1. A similar inhibition of ZIKV binding to Aag-2 cells, with a 2-fold (46%) reduction, was also observed when *w*AlbB density was high. Previous studies reported that *w*Stri inhibited entry of ZIKV into *A. albopictus* cells, while a similar inhibition was not observed in *w*Mel-infected Aag-2 cells (Schultz et al., 2018; Thomas et al., 2018). One of the potential reasons for these inconsistent observations is that the cell lines used in the two former studies may have had different *Wolbachia* densities, similar to the different results that were observed here in the ZIKV binding assays, using the same cell line but with either a low or high density of *w*AlbB. Consistent with a reduction in viral binding to W-Aag-2 cells, more viral particles were present in the culture medium of W-Aag-2 compared to that of R-Aag-2 cells. Furthermore, although they did not effectively bind to W-Aag-2 cells, using a plaque assay, we were able to demonstrate that unbound DENV-2 virions exposed to W-Aag-2 cells remained infective to mosquito cells lacking *Wolbachia* infection (Figure 2B). This latter result would seem to refute the hypothesis that antiviral effectors secreted into the culture medium from W-Aag-2 cells inactivate of DENV and thereby inhibit its binding to W-Aag-2 cells. There was a ~6-fold reduction in the copy number of DENV-2 at 1 h post-infection in W-Aag-2 compared to R-Aag-2 cells at 25°C, while only ~3-fold reduction was observed at 4°C. As the level of viral infection was similar in W-Aag-2 cells at both temperatures, this variation might be due to initiation of viral RNA replication following the binding stage in R-Aag2 cells at 25°C. This is consistent with the previous observations that DENV enters into mosquito cells within 5–7 min, and its replicative intermediate RNA could be detected as soon as 20 min post-infection in mosquito cells (Vaughan et al., 2002; Mosso et al., 2008).

In order to understand the molecular mechanism by which *w*AlbB inhibited the binding of DENV to mosquito cells, we determined the relative expression and knocked-down host genes, previously reported as being bound by DENV. Our results indicate that binding of DENV to cells may be inhibited by *Wolbachia* through its suppression of the expression of two membrane proteins: dystroglycan and tubulin, but it did not support the involvement of those *w*AlbB-induced host membrane proteins, including cadherin or enolase, in inhibition of viral binding to Aag-2 cells. The former is consistent with previous evidence of direct interaction between these putative DENV receptors. Alpha-dystroglycan is an extra-cellular protein which binds to laminin, a component of the extracellular matrix, and to beta-dystroglycan, a transmembrane protein which binds to components of the cytoskeleton, including tubulin and actin. Direct binding of the laminin receptor to tubulin and actin was also reported previously (Venticinque et al., 2011). Interestingly, actin is also downregulated by *Wolbachia*, and previous studies have showed that actin is involved with viral endocytosis and replication (Acosta et al., 2008). In both filarial nematodes and *Drosophila*, *Wolbachia* has been shown to interact with the cytoskeletal proteins, actin and tubulin, with potential functions involving facilitation of bacterial migration, distribution, and maternal transmission (Ferree et al., 2005; Melnikow et al., 2013). Thus, interaction of *Wolbachia* with a molecular complex comprising dystroglycan, tubulin, and actin may decrease the binding of DENV to mosquito cells.

It should be noted that the magnitude of viral binding inhibition is lower in RNAi-mediated silencing of either dystroglycan or tubulin than observed in the W-Aag-2 cells, although the degree of silencing produced by dsRNA is higher than the downregulation produced by *w*AlbB. This is probably caused by silencing of only one single gene in the dsRNA treatment, whereas *w*AlbB suppresses numerous host genes simultaneously, which can produce additive or synergistic effects on inhibition of viral binding to host cells. Viruses may utilize multiple redundant host membrane proteins to mediate the binding such that silencing of a single host gene may cause only a subtle effect on inhibition. In addition, transient effects induced by dsRNA-mediated gene silencing may also limit the robustness of this approach in recapitulating the ability of *Wolbachia* to affect those viral host factors during its persistent intracellular infection. However, it is possible that there are other unknown factors involved in this *Wolbachia*-mediated inhibition of viral binding to host cells. Lack of inhibition of ZIKV binding to Aag-2 cells at a low level of *w*AlbB infection suggests that the intracellular *Wolbachia* titer should be above a threshold to interfere with viral binding to the cells. Although further studies are needed to fully elucidate the impact of *Wolbachia* on each stage in the virus life cycle, the previous observation of viral inhibition without impact on viral binding (Thomas et al., 2018) suggests that *Wolbachia*-mediated viral inhibition may be mainly exerted in intracellular replication with the binding as an additional step.

The presence of negative-strand RNA is a hallmark of DENV replication within host cells. In order to study the impact of *Wolbachia* on viral replication, we used a transfection assay to directly introduce the DENV-2 genome into the cytoplasm of host cells and so bypass the initial life cycle stages of viral binding, entry, and uncoating. We then measured the copy number of viral negative-strand RNA. Even though an equivalent amount of DENV-2 genome was introduced into both W-Aag-2 and R-Aag-2 cells, we observed significantly lower copy numbers of viral negative-strand RNA in W-Aag-2 cells as compared to R-Aag-2 cells. Since it takes about 1 day for DENV to start *de novo* virion production in Aag-2 cells (Sim and Dimopoulos, 2010), the negative-strand RNA at 4 h post-transfection should come only from the initial round of replication of the primarily infecting virus. Thus, this result provides direct evidence that *w*AlbB can inhibit viral replication even after viral entry into host cells. Furthermore, we observed an increase in viral inhibition with increasing time after transfection. At days 3 and 7 post-transfection, there was significantly less negative-strand RNA in W-Aag-2 cells than in R-Aag-2 cells. The viral titer was also significantly lower in the supernatant of W-Aag-2 cells than in that of R-Aag2 cells at 5 days post-transfection. However, this difference at later time points could be caused by both inhibition of binding and replication because progeny viruses can be subject to interference at both stages.

Overall, our findings highlight several important aspects for understanding both the mechanism and practical application for disease control of *Wolbachia*-mediated viral interference. First, *Wolbachia*-mediated viral inhibition occurs at multiple stages of the DENV life cycle, including binding and replication, resulting in a high efficacy of blocking viral propagation. With up to 75% reduction in DENV binding to host cells, this could be one of the important factors contributing to the overall outcome of viral interference. Targeting DENV at multiple stages of its life cycle would also make it more difficult for DENV to evolve resistance to *Wolbachia* than other antiviral agents, which target only a single stage in the viral life cycle. It is worth noting that *w*AlbB also induced inhibition of ZIKV binding to mosquito cells, indicating that the viral interference associated with *w*AlbB is a broad spectrum. Second, like antiviral drugs, *Wolbachia* does not appear to destroy either the viral genome or assembled infectious virions; instead, *Wolbachia* inhibits progression through the viral life cycle preventing the formation of new virus. Given our current lack of anti-dengue drugs, understanding the mechanism of *Wolbachia*-mediated viral inhibition may provide insights into the rational design and development of new drugs for medical therapy. Finally, *Wolbachia*-mediated viral interference occurs through alteration of host factors that are required for viral growth. Future studies should continue focusing on identification and characterization of the host factors that interact with *Wolbachia* to inhibit viral binding and replication. This knowledge may contribute toward and facilitate the development of novel strategies for the control of mosquito-borne diseases.

## Data Availability Statement

All datasets presented in this study are included in the article/Supplementary Material.

## Author Contributions

PL and ZX conceived the idea, designed the experiments, and supervised the project. PL and QS performed the majority of the experiments and analyzed the data. PF, KL, and XL performed partial experiments. PL, QS, and ZX wrote the manuscript. All authors contributed to the article and approved the submitted version.

### Conflict of Interest

ZX was employed by the Guangzhou Wolbaki Biotech Co., Ltd.

The remaining authors declare that the research was conducted in the absence of any commercial or financial relationships that could be construed as a potential conflict of interest.
