# Supplementary material for: Wolbachia Inhibits Binding of Dengue and Zika Viruses to Mosquito Cells
Source: Front Microbiol. 2020 Aug 4;11:1750. doi: 10.3389/fmicb.2020.01750 (PMC7417768; doi:10.3389/fmicb.2020.01750)
Supplement: Supplementary file 1 [file Data_Sheet_1.docx]

**Table S1. List of primer sequences used for analysis of transcription.**

| Gene ID | Forward 5’– 3’ | Reverse 5’– 3’ |
| --- | --- | --- |
| AAEL003863 | GTCAAGCGTATCTCCGGACT | GTGTAGGTAACGGCATCACG |
| AAEL015390 | TTCACCGACACCTGAAGAAC | CTAGGATGGCAGCGGAATA |
| AAEL002851 | ACGTCTGCACTTCTTCATGC | ATCGAACATCTGTTGGGTCA |
| AAEL017096 | TCGTAATTGGACACGTCGAT | CTTGAAGGAACCCTTACCCA |
| AAEL003670 | GGTTTCGCAGTCGTTGAGTA | CGGTTAAGCTCACCCTTCTC |
| AAEL001928 | GCTGATCGTATGCAGAAGGA | CCGATCCAGACGGAGTATTT |
| AAEL001477 | AGAAGCGAGCCAATCGTAGT | TGCTGGTGTTGTTGTTGTTG |
| AAEL013147 | AGAAGCGAGCCAATCGTAGT | TGCTGGTGTTGTTGTTGTTG |
| AAEL015681 | GCATCATGAACAGCTTCGTC | AGCTGGTGTATTTGGTGACG |
| AAEL009994 | TTCCAAGAAACGCAAGTACG | AACTTGAAGGCGGTGTTCTT |
| AAEL006868 | AGTGCCGATTGGTTCAGTTT | CGATTTCGTGTTTGTCCTTG |
| AAEL009345 | GTCGACGCTGCATTAGAGAA | GGATGTTTCAACTTAATGATCGC |
| AAEL000032 | TCGCTGTGCACTTAATCCTC | AGAGACCAAAGCTGGAAGGA |
| AAEL007439 | CTGGATCAACGGCTCATAAA | AGTTAAACTGAGGCCGCAAC |
| AAEL001668 | ACAACGTCAAGGCCGACT | AGTCCCGAGTTCAGCACAG |
| AAEL001411 | CGCAGAAAGTGAGTGTCCAG | AACAACAGCGAACAACGAAA |
| AAEL001196 | ACCGTTAACGACGATGATGA | GGTTGAACCACCCTCAAACT |
| AAEL000386 | TCTTGCTCGAAACGCTAGAA | TTCACAGCTTGCGTTACTCC |
| AAEL003594 | ACTCACAAGATGATGCCCAA | AGAGCTCGTGTTGCAGCTTA |
| AAEL003827 | GGACTTCAAGACCGATCTGC | GCGCAAAGGTTGGTATCTTC |
| DQ440299 | TCCTGCACTTCTTCGGACTT | TACAGTTGCGTGGGTGTGTT |

**Table S2. List of primer sequences used for RNAi silencing and measurement of transcription knockdown efficacy.**

| Gene ID | Primer Name | Primer sequence (5’– 3’) | |
| --- | --- | --- | --- |
| AAEL002851 | Tub-RNAi-F | | TAATACGACTCACTATAGGGACTAGTCCCCTTCAGAGCAGC |
|  | Tub-RNAi-R | | TAATACGACTCACTATAGGGGCCGGAGGCTTCATTGTAGT |
|  | Tub-effi-F | | TGCTAGTCGATCTGGAACCC |
|  | Tub-effi-F | | CCTCGGTGTAGTGTCCCTTG |
| DQ440299 | HSC70-RNAi-F | | TAATACGACTCACTATAGGGATCATGGACAAGTGCAACGA |
|  | HSC70-RNAi-R | | TAATACGACTCACTATAGGGAGTCGACTTCCTCGATGGTG |
|  | HSC70-effi-F | | AGACGAGAAGCAGAAGGAAAC |
|  | HSC70-effi-F | | GTATCGTTGCACTTGTCCATG |
| AAEL001477 | Lam-RNAi-F | | TAATACGACTCACTATAGGGATACAAGTGGCAAGCCAACC |
|  | Lam-RNAi-R | | TAATACGACTCACTATAGGGTCACAGGTGTCACCGAATGT |
|  | Lam-effi-F | | AATGCGGATTGTGTCATCAT |
|  | Lam-effi-F | | ATGCGCACTTCTTACAGTCG |
| AAEL013147 | Dys-RNAi-F | | TAATACGACTCACTATAGGGGCAGGCTACTGATTCCGGT |
|  | Dys-RNAi-R | | TAATACGACTCACTATAGGGCATTGAGGACTTTTTCGGTCA |
|  | Dys-effi-F | | CCAGGATCCAAACCTTGCTA |
|  | Dys-effi-F | | CTGTCCGATTTGTTGTCCCT |
| AAEL006868 | AKR-RNAi-F | | TAATACGACTCACTATAGGGTGTGCTTCATCCTGGATCTG |
|  | AKR-RNAi-R | | TAATACGACTCACTATAGGGTGTCCCGTTGTAGCAGTCCT |
|  | AKR-effi-F | | GCGAATCAAAATGAAGCAGGG |
|  | AKR-effi-F | | CAGATCCAGGATGAAGCACAG |
| AAEL009345 | Proh-RNAi-F | | TAATACGACTCACTATAGGGCTACCAGATGAGCCGGTCC |
|  | Proh-RNAi-R | | TAATACGACTCACTATAGGGAATTCAGCGATCAGCAAACC |
|  | Proh-effi-F | | GGTGATGGCTTGGTTGAACT |
|  | Proh-effi-F | | TACTGTGGCAACTGGAGCAG |
| AAEL001668 | Enol-RNAi-F | | TAATACGACTCACTATAGGGGTCGGTGATGAGGGTGGTT |
|  | Enol-RNAi-R | | TAATACGACTCACTATAGGGGTGTTGGCAGTCATCTTTGC |
|  | Enol-effi-F | | ACCACCACCTGAAGAACGTC |
|  | Enol-effi-F | | ATCAGGTTCAGGGCTTCCTT |
| AAEL001196 | Cadh-RNAi-F | | TAATACGACTCACTATAGGGCTTCGACATCAACAAAGCGA |
|  | Cadh-RNAi-R | | TAATACGACTCACTATAGGGTTCCCATCAGTTTCCTCGAC |
|  | Cadh-effi-F | | TGAACGATATGCCTCCACAT |
|  | Cadh-effi-F | | CGGTTACTGTGAGAATTGGC |
